# Effect of retirement on self-rated oral health and dental services use: longitudinal fixed-effects instrumental variable study in 31 countries

**DOI:** 10.5271/sjweh.4134

**Published:** 2024-03-01

**Authors:** Sebastian-Edgar Baumeister, Prof Dr, Hanna Wesselmann, Assistant Researcher, Gustavo G Nascimento, Stefan Listl, Prof Dr

**Affiliations:** 1 Institute of Health Services Research in Dentistry, University of Münster, Münster, Germany.; 2 National Dental Research Institute Singapore, National Dental Centre Singapore, Singapore.; 3 Oral Health Academic Programme, Duke-NUS Medical School, Singapore.; 4 Radboud Institute of Health Sciences (RIHS), Department of Dentistry, Quality and Safety of Oral Healthcare, Radboud University Medical Center, Nijmegen, The Netherlands.

**Keywords:** dental service, endogeneity, oral health, population aging, retirement

## Abstract

**Objective:**

This study examined the effect of retirement on self-rated oral health and dental services use.

**Methods:**

Covering 31 countries, we used harmonized panel data from the English Longitudinal Study on Aging (ELSA), Health and Retirement Study (HRS), and the Survey of Health, Aging and Retirement in Europe (SHARE). Data comprised 485 085 observations from 112 240 individuals aged ≥50 years. Official and early retirement ages were leveraged as instruments in a fixed-effects instrumental variable approach.

**Results:**

We found that retirement exhibited a negative effect on self-rated oral health (β = -0.37; 95% confidence interval (CI) -0.44– -0.30) and a positive effect on the propensity to seek dental care (β = 0.56; 95% CI 0.53–0.60). Male retirees showed a stronger decrease in self-rated oral health and increase in dental services use than female retirees. Participants who previously worked in a physically demanding job showed a stronger effect on self-rated oral health. Conversely, participants without a physically demanding job in the past exhibited a stronger retirement effect on dental service use. Compared with other health system clusters, retirement effects on dental services use were stronger in the health system clusters (iii) Belgium, Denmark, Finland, France, Ireland, Luxembourg, The Netherlands, Sweden, (vi) Israel and (vii) the United States.

**Conclusions:**

Using a quasi-experimental design, we found that transition to retirement lowers self-rated oral health and increases the use of dental services. Retirement effects appeared heterogeneous across sexes, type of previous labor, and health systems.

Population aging is a major public health challenge. By 2030, 1 in 6 people in the world will be ≥60 years. High-income countries are raising the retirement age to counter the effects of population aging (1). Transition to retirement is a major life event that can also have significant impact on late-life well-being and health and lead to both beneficial and detrimental outcomes. Changes in retirement age may affect health and healthcare consumption of older individuals and, therefore, impact fiscal spending. A growing body of research investigated the effects of retirement on health, focusing mainly on self-rated health, health services utilization, and mortality (2–7). However, in these studies, measurement instruments for self-rated health did not cover oral health, and healthcare services excluded dental care. The present study adds to the literature by investigating the effects of retirement on self-rated oral health and dental services utilization. A methodological challenge in empirical studies of retirement is endogeneity (7). There might be unadjusted common causes of retirement and health outcomes causing residual confounding. A decline in health status may affect the decision to retire and induce reverse causation.

We applied two causal identification strategies to estimate the effect of retirement and address unobserved confounding and reverse causation. We used fixed-effects modeling for estimating causal effects in panel data because they flexibly control for unobserved time-invariant confounding (8). We combine fixed-effects modeling with a design-based instrumental variable strategy to further account for unobservable time-varying characteristics. Conceptually, the instrumental variable design shares characteristics with an experimental randomized study (9, 10). To draw causal inferences, both designs rely on two essential assumptions. First, the exogenous factors – randomization or a social policy – induce variations in the likelihood of treatment allocation among sets of individuals sharing otherwise identical potential outcomes. Second, the exogenous factors must have no other mechanisms influencing the outcome effect via the treatment. If these assumptions are met, the statistical associations between the exogenous factors or instrument, the treatment, and the outcome can be leveraged to estimate the causal effect of treatment on the outcome. Instruments can take the form of arbitrary discontinuities, such as an arbitrary cutoff for eligibility for statutory or early retirement (7, 11). The instrumental variable approach leverages discrete change in retirement probability at an eligibility age. Official and early retirement ages create differences in realized retirement between individuals who are otherwise similar. Exploiting multicounty cohort studies and a fixed-effects instrumental variable method, the current study aimed to estimate the effect of retirement on self-rated oral health and dental services use. We also examined whether retirement effects are heterogenous across relevant population subgroups and health systems.

## Methods

### Cohort studies and study participants

Harmonized panel data provided by the Gateway to Global Aging Data network (12) came from the English Longitudinal Study on Aging (ELSA), Health and Retirement Study (HRS), and the Survey of Health, Aging and Retirement in Europe (SHARE). ELSA is a nationally representative cohort study of ≥50-year-old adults living in England and interviewed every two years since 2002 (nine waves have been collected) (13). The London Multicentre Research Ethics Committee (MREC/01/2/91) approved ELSA. HRS is a representative cohort study that has been surveying community-dwelling ≥50-year-old North Americans every two years since 1992 (15 waves) (14). The Institutional Review Board at the University of Michigan and the National Institute on Aging (HUM00061128) approved HRS. SHARE is a cross-national cohort study of ≥50-year-old adults across 29 countries that has been collecting data every two years since 2004 (8 waves) (15). The Ethics Committee at the University of Mannheim and the Ethics Council of the Max-Planck Society have both approved the SHARE study. Additionally, country-specific ethics committees or institutional review boards approved implementations of SHARE in the participating countries. Informed consent was obtained from all participants in all cohorts. The analytical datasets were restricted to eligible participants aged ≥50 years at baseline who were in the labor force in any capacity at the initial wave and remained in the study for at least one subsequent follow-up interview. Using ELSA, we created a longitudinal dataset consisting of 25 185 observations from 11 519 individuals to study the effect of retirement on self-rated oral health. We pooled HRS and SHARE to generate a longitudinal dataset of 459 900 observations from 100 721 individuals participating in up to 15 waves to estimate the effect of retirement on dental services use.

### Outcomes, retirement, instruments, and covariates

Self-rated oral health was assessed using one item with a Likert-type scale [“poor” (1) to “excellent” (5)] in ELSA waves three, five, seven, eight and nine (table 1). Dental services use was defined as ≥1 visit to a dentist over the previous two years in each HRS wave and ≥1 annual visit to a dentist in each SHARE wave (table 2). The supplementary material (www.sjweh.fi/article/4134), tables S1–4 provide descriptive information for both outcome variables and covariates stratified by sex. Supplementary tables S5–34 displays descriptive statistics by country in pooled HRS and SHARE data. Respondent’s labor force and retirement status at each wave were assessed using information from subjective retirement status and labor force status. Respondents were classified as retired if they were not working and reported being retired, and compared to workers. We instrumented retirement using official and early retirement ages (11, 7). Dummy variables for official and early retirement ages indicated whether participants had attained the age to be entitled to official and early retirement, respectively. Data for official and early retirement ages were compiled for the study periods (supplementary table S35) (16, 1).

**Table 1:** Descriptive statistics of observations by labor force status in the English Longitudinal Study on Aging (ELSA).

		Working (obs.=7655)			Retired (obs.=17 530)	
		N (%)	Mean (SD)		N (%)	Mean (SD)
Self-rated oral health						
	Poor	353 (4.6)			960 (5.5)	
	Fair	1145 (15.0)			2562 (14.6)	
	Good	2939 (38.4)			7101 (40.5)	
	Very good	2175 (28.4)			4923 (28.1)	
	Excellent	1043 (13.6)			1984 (11.3)	
Age, years			61.9 (5.4)			73.7 (7.7)
Men		2782 (50.7)			6248 (45.3)	
Married		4086 (74.5)			8707 (63.1)	
Education						
	Low	780 (15.3)			3726 (29.5)	
	Middle	2884 (56.5)			6452 (51.1)	
	High	1439 (28.2)			2443 (19.4)	
Type of previous work						
	No physically demanding work	5063 (66.1)			-	
	Physically demanding work	2592 (33.9)			-	
	Current smoking	596 (10.9)			932 (6.8)	
Body mass index (kg/m^2^)						
	<30	426 (27.7)			1275 (30.6)	
	25–30	628 (40.8)			1696 (40.8)	
	>30	486 (31.6)			1189 (28.6)	
Diabetes		500 (9.1)			1921 (13.9)	

**Table 2:** Descriptive statistics of observations by labor force status in pooled the Health and Retirement Study (HRS) and the Survey of Health, Aging and Retirement in Europe (SHARE).

		Working (observations=183 523)			Retired (observations=276 377)	
		N (%)	Mean (SD)		N (%)	Mean (SD)
Dental services use		97 817 (53.3)			186 554 (67.5)	
Age, years			59.9 (6.7)			73.2 (8.4)
Men		90 318 (49.2)			130 190 (47.1)	
Married		129 552 (70.6)			170 774 (61.8)	
Education						
	Low	37 621 (20.5)			106 272 (38.5)	
	Middle	65 729 (35.8)			95 538 (34.6)	
	High	80 158 (43.7)			74 546 (27.0)	
Type of previous work						
	No physically demanding work	135 399 (73.8)			-	
	Physically demanding work	48 124 (26.2)			-	
Dental coverage		47 704 (26.0)			48 285 (17.5)	
Current smoking		26 968 (15.1)			237 74 (8.8)	
Body mass index (kg/m^2^)						
	<30	49 796 (32.4)			82 028 (35.4)	
	25–30	62 993 (41.0)			94 862 (41.0)	
	>30	40 870 (26.6)			54 746 (23.6)	
Diabetes		22 050 (12.0)			56 303 (20.4)	

The modified disjunctive cause criterion and Anderson’s Behavioral Model of Health Services Utilization were applied to select covariates to adjust for time-varying confounding. The disjunctive cause criterion chooses direct causes (or proxies of causes) of the exposure or outcome to derive a sufficient set of (time-varying) confounders (17). The Anderson model of access to care is one of the most widely used and accepted models of access in the health services research literature (18). Access is conceptualized as a function of the need for healthcare as indicated by the presence of symptoms or illness; enabling factors, such as income and health insurance status, which allow individuals to satisfy a need for care; and predisposing factors, which reflect preferences, styles of healthcare use, expectations, and other non–health-related factors that affect the demand for care. We investigated whether retirement effects varied across subgroups (sex, type of previous work, healthcare systems). Health systems were grouped into seven clusters, following Ferreira et al (19): (i) Austria, Germany, Switzerland; (ii) Belgium, Denmark, and Finland, France, Ireland, Luxembourg, The Netherlands, and Sweden; (iii) Cyprus, Greece, Italy, Malta, Portugal, and Spain; (iv) Bulgaria, Hungary, Latvia, Lithuania, Slovakia, and Romania; (v) Croatia, Czech, Estonia, Poland, and Slovenia; (vi) Israel; and (vii) the United States.

### Statistical analyses

We estimated the effect of retirement on self-rated oral health and dental services utilization using a linear probability instrumental variable model implemented by fixed-effects (FE) two-stage least-squares estimation (FEIV) (8). The FE method accounts for time-invariant unobserved person characteristics that are correlated with both treatment and outcome variables and time FE to control for time-specific shocks across panel waves. As two-way FE modeling is based on within-person variation, all time-constant covariates (eg, sex, education) are implicitly controlled (8). We modeled retirement as a second-order lag to preserve the temporal order of treatment and outcomes. To account for time-varying confounders, we adjusted the models for self-rated oral health for age, marital status, smoking, body mass index, and diabetes (table 1). The models for dental service use included age, marital status, smoking, body mass index, and dental coverage (table 2). Time- and individual-level FE do not account for unobserved time-varying confounds, such as decline in health status between panel waves, that could induce retirement. Thus, we leveraged official and early retirement age as instruments for retirement. At the first stage of the two-stage least-squares model, we predicted retirement using official and early retirement age and their interactions with age and age-squared, along with additional time-varying covariates. We compared estimates from standard FE and FEIV models. We additionally estimated a FE-ordered logit model for self-rated health and a FE Poisson model for dental services use with instrumental variables using a control function approach (20, 21). Heterogenous effects across subgroups (sex, type of previous work, health system clusters) were examined using a correlated random-coefficient instrumental variable (CRC) model (22). The CRC approach is an extension of the IV method to estimate the conditional average treatment effect in a model in which multiple treatments are present. It employes a correction function approach to control for endogeneity driven by unobserved heterogeneity that may simultaneously affect the treatment and the modifier. We applied cluster-robust standard errors, survey weights to account for the complex sampling, and inverse probability weighting to adjust for panel attrition (8). Analyses were performed using R 4.3.1 (R Foundation for Statistical Computing, Vienna, Austria) and Stata 17 (Stata Corporation, College Station, TX, USA). The reporting was based on recommendations by STROBE (STrengthening the Reporting of OBservational studies in Epidemiology).

## Results

### Main results

In a FE model, retirement was associated with a decrease in self-rated oral health (β=-0.14; 95% confidence interval (CI) -0.17– -0.10) (table 3). The FEIV model showed a -0.37-point (95% CI -0.44– -0.30) decrease in self-rated oral health due to retirement. The FE model showed that retirement increased the risk of dental service use (β=0.03; 95% CI 0.03–0.04). The FEIV model showed a 0.56 increase (95% CI 0.53–0.60) in the risk of dental services use. First-stage F statistics were >50, suggesting strong instruments, and did not differ substantially across countries. Over-identification tests showed that instruments were uncorrelated with residuals.

**Table 3:** Effects of retirement on self-rated oral health and dental services use. [FE=fixed-effects linear probability model; FEIV=fixed-effects instrumental variable linear probability model; CI=confidence interval. β=estimate for average treatment effect from FE model and local average treatment effect from FEIV model. $\frac{d\left(\ln_{y}\right)}{\mathrm{dx}}$ = semielasticity.]

Outcome	Model	β	95% CI for β	$\frac{d\left(\ln_{y}\right)}{\mathrm{dx}}$	95% CI for $\frac{d\left(\ln_{y}\right)}{\mathrm{dx}}$
Self-rated oral health ^a^	FE	-0.135	-0.174– -0.097	-0.041	-0.53– -0.029
	FEIV	-0.366	-0.437– -0.295	-0.112	-0.134– -0.090
Dental services use ^b^	FE	0.033	0.026–0.040	0.076	0.060–0.0923
	FEIV	0.563	0.525–0.601	1.304	1.216–1.392

^a^ Models for self-rated health were adjusted for age, age^2^, marital status, smoking, body mass index, and diabetes. ^b^ Models for dental services use included age, age^2^, marital status, smoking, body mass index, and dental coverage.

### Heterogenous effects and robustness checks

We found that effects of retirement on self-rated oral health and dental services use were heterogeneous across subgroups in CRC models (table 4). The effect of retirement on self-rated oral health was stronger in men and in participants retiring from physically demanding work. The effect of retirement on dental services use was stronger among men, participants without physically demanding work in the past, and in health system clusters (ii), (iv), and (vii). Results from control function FE-ordered logit model and control function FE Poisson model were consistent with the primary FEIV analyses (supplementary tables S36 and S37).

**Table 4:** Heterogenous effects of retirement on self-rated oral health and dental services use by subgroup in correlated random-coefficient instrumental variable models.^a^ [CI=confidence interval.]

			β	95% CI
Self-rated oral health				
	Men		-0.450	-0.570– -0.331
	Women		-0.344	-0.436– -0.252
	No physically demanding work		-0.325	-0.378– -0.271
	Physically demanding work		-3.734	-5.772– -1.695
Dental services use ^b^				
	Men		0.537	0.497–0.577
	Women		0.466	0.433–0.498
	No physically demanding work		0.510	0.474–0.546
	Physically demanding work		0.284	-0.180–0.748
	Health system clusters			
		Austria, Germany, Switzerland	0.134	0.107–0.162
		Belgium, Denmark, Finland, France, Ireland, Luxembourg, The Netherlands, and Sweden	0.205	0.176–0.234
		Cyprus, Greece, Italy, Malta, Portugal, and Spain	0.154	0.114–0.194
		Bulgaria, Hungary, Latvia, Lithuania, Slovakia, and Romania	0.017	-0.055–0.089
		Croatia, Czech, Estonia, Poland, Slovenia	0.091	0.062–0.119
		Israel	0.203	0.104–0.302
		United States	0.390	0.364–0.416

^a^ Correlated random-coefficient instrumental variable models for self-rated health adjusted for sex, age, age^2^, education, marital status, smoking, body mass index, and diabetes. ^b^ The models for dental services use adjusted for sex, age, age^2^, education, marital status, smoking, body mass index, and dental coverage.

## Discussion

Taking advantage of multinational longitudinal data, this study estimated the effect of retirement on self-rated oral health and dental services use by means of two complementary empirical identification strategies: the analytical two-way fixed-effects strategy to absorb time-invariant confounding factors and the design-based instrumental variable strategy to account for unobserved time-varying confounding and reverse causation. Both empirical approaches could be subject to bias but the bias is expected to be independent (10, 23). Our results show that retirement has detrimental effects on self-rated oral health and leads to an increase in the propensity to seek dental care.

Recent studies have examined the effects of retirement on general self-rated health indicators and outpatient visits to general practitioners and specialists with mixed results. Previous studies reported a decrease in self-rated health after retirement, while other found an increase in self-rated health (2, 24–30, 5). A recent meta-analysis pooled estimates from 47 studies and found a negligible effect of retirement on general self-rated health (6). Most of the studies on the impact of the transition from employment to retirement found a decrease in doctor visits (4, 26, 31). While few studies found no change in doctor visits (3, 5), others found an increase (32, 33). Various factors could explain these seemingly contradictory findings, especially the application of different research designs to different populations in different countries (5). Nishimura et al (34) and Garrouste & Perdrix (7) investigated which factors explain differences in empirical studies investigating the effect of retirement on health. Both groups found that the retirement definition and country played no role, but that differences in health measurement and identification strategy produced different study findings.

Several mechanisms could explain a decrease in subjective oral health and increase in dental care after the transition from employment to retirement (35, 36). As pension is generally lower than previous earnings, reduced income after retirement can produce a change in budget constraints. Change in budget constraints after retirement can also be due to change in insurance coverage and increase in (oral) healthcare costs. Income reduction could decrease (oral) health through change to a more affordable but less healthy diet, unhealthy lifestyle, lower living conditions and reduced access to care. Change in the marginal benefit of health may arise if there are changes in utilities associated with good health (35, 36). Following this model, the marginal benefits of (oral) health might decrease after retirement. Another explanation for subjectively perceived worsened oral health might be that retirement leads to more time available to reflect on (oral) health, thus becoming more aware of previously not recognized (oral) health issues. The observed increase in dental care following retirement might be due to having more time available to visit the dentist, or it could be triggered by (subjectively perceived) worsened oral health after retirement.

We found that effects of retirement on self-rated oral health and dental services use were heterogenous. Retirement effects were stronger among men than women. This gender disparity could be due to higher oral health awareness of women during work life and, consequently lower oral health needs post-retirement or caused by gender differences in employers’ willingness to grant time off work for employees to visit a dentist (3). Although the analyses were limited to those who had working experience during the study period, people retiring from physical labor exhibited worse self-rated health and lower risk of dental services use. Participants working in a physical demanding job might have had lower earnings, and it is well established that lower income negatively impacts oral health (37). Lower income after retirement might reduce dental coverage, making physical laborers less likely to seek dental care. The strongest effects on dental services use were seen in the health system clusters ‘Belgium, Denmark, Finland, France, Ireland, Luxembourg, The Netherlands, and Sweden’, ‘Israel’, and the ‘United States’. However, the direction of effect estimates on dental services use were consistent across health systems, ensuring robustness of results (23). In the United States, the more pronounced increase in dental services use after retirement could be due to a ‘lag effect’ whereby new retirees seek dental services before losing (full) dental coverage after transitioning to retirement.

This study has several limitations. First, we did not separate type of retirement (eg, statutory, early, involuntary, health-related) and type of dental services (preventive or curative). Second, we used a simple measure to assess self-rated oral health that might not be as valid and reliable as a multi-dimensional measure. Third, retirement status and outcomes were self-reported, which might be subject to measurement errors. Yet, brief assessments of self-reported oral health are strongly correlated with objective deterioration in oral health (38). Forth, the instrumental variable approach allowed us to estimate the short-term impact of the transition from employment to retirement. This strategy is not suitable for studying the long-term effects of retirement on oral health or dental services use. To estimate long-term effects, the instrument would need to vary both within and between cohorts to separate the time spent in retirement from aging. Fifth, we grouped countries according to a recently proposed classification of health systems. There are several health system classifications, and it is possible that another classification would have produced different results. Last, although we used data from several European countries, Israel and the United States, effects might differ in other regions and countries; particularly in low- and middle-income countries. Instrumental variable regression estimates a local average treatment effect that might not generalize to the entire population. However, heterogeneous effect modeling using a CRC model provides a conditional average treatment effect applicable to a specific subgroup.

In conclusion, this study suggests that retirement results in deteriorated self-rated oral health and increases use of dental services. Additional studies are warranted that examine whether effects vary across types of retirement and dental services with data sources containing objective measures of oral health and dental care consumption. Future studies could also examine the oral effects of retirement in other regions and health systems and determine the mechanisms underlying the changes in oral health and dental services use after retirement.

## Supplementary material

Supplementary material
