# Supplementary material for: Effect of retirement on self-rated oral health and dental services use: longitudinal fixed-effects instrumental variable study in 31 countries
Source: Scand J Work Environ Health. 2024 Feb 28;50(2):96–102. doi: 10.5271/sjweh.4134 (PMC10927317; doi:10.5271/sjweh.4134)
Supplement: Supplementary material [file SJWEH-50-96-S001.pdf]

Effect of retirement on self-rated oral health and dental services use: longitudinal fixed-effects instrumental variable study in 31 countries[1]

by Sebastian-Edgar Baumeister, Prof Dr,[2] Hanna Wesselmann, Assistant Researcher, Gustavo G Nascimento, DMD, PhD, Stefan Listl, Prof Dr

1. Supplementary material
2. Correspondence to: Sebastian-Edgar Baumeister, Institute of Health Services Research in Dentistry, University of Münster, Albert-Schweitzer-Campus 1, 48149 Münster, Germany. [E-mail: sebastian.baumeister@uni-muenster.de]

Supplementary Table S1 Descriptive statistics of male participants by labor force status in ELSA

	Working (obs. = 2782)	Retired (obs. = 6,248)
Self-rated oral health		
Poor	183 (6.6%)	423 (6.8%)
Fair	446 (16.0%)	1004 (16.1%)
Good	1048 (37.7%)	2500 (40.0%)
Very good	759 (27.3%)	1610 (25.8%)
Excellent	346 (12.4%)	711 (11.4%)
Age, years	63.6 (5.5)	74.0 (7.4)
Married	2237 (80.4%)	4643 (74.3%)
Education		
Low	379 (14.4%)	1404 (23.8%)
Middle	1429 (54.4%)	3086 (52.3%)
High	817 (31.1%)	1409 (23.9%)
Type of previous work		
No physically demanding work	1832 (65.9%)	-
Physically demanding work	950 (34.1%)	-
Current smoking	273 (9.8%)	424 (6.8%)
Body mass index		
<30	178 (22.7%)	498 (26.6%)
25-30	358 (45.7%)	882 (47.2%)
>30	247 (31.5%)	490 (26.2%)
Diabetes	325 (11.7%)	1013 (16.2%)

ELSA = English Longitudinal Study on Aging

Supplementary Table S2 Descriptive statistics of female participants by labor force status in ELSA

	Working (obs. = 2706)	Retired (obs. = 7543)
Self-rated oral health		
Poor	110 (4.1%)	392 (5.2%)
Fair	409 (15.1%)	1085 (14.4%)
Good	1047 (38.7%)	3058 (40.5%)
Very good	772 (28.5%)	2204 (29.2%)
Excellent	368 (13.6%)	804 (10.7%)
Age, years	61.8 (5.1)	73.6 (7.8)
Married	1849 (68.3%)	4064 (53.9%)
Education		
Low	401 (16.2%)	2322 (34.5%)
Middle	1455 (58.7%)	3366 (50.1%)
High	622 (25.1%)	1034 (15.4%)
Type of previous work		
No physically demanding work	1817 (67.1%)	-
Physically demanding work	889 (32.9%)	-
Current smoking	323 (11.9%)	507 (6.7%)
Body mass index		
<30	248 (32.8%)	777 (33.9%)
25-30	270 (35.7%)	814 (35.5%)
>30	239 (31.6%)	699 (30.5%)
Diabetes	175 (6.5%)	908 (12.0%)

ELSA = English Longitudinal Study on Aging

Supplementary Table S3 Descriptive statistics of male participants in pooled HRS and SHARE by labor force status

	Working (obs. = 90318)	Retired (obs. = 130190)
Dental services use	45882 (50.8%)	82801 (63.6%)
Age, years	60.6 (7.0)	73.1 (8.2)
Married	70355 (77.9%)	98674 (75.8%)
Education		
Low	19785 (21.9%)	50352 (38.7%)
Middle	31552 (34.9%)	43515 (33.4%)
High	38968 (43.2%)	36306 (27.9%)
Type of previous work		
No physically demanding work	66027 (73.1%)	-
Physically demanding work	24291 (26.9%)	-
Dental coverage	22373 (24.8%)	22549 (17.3%)
Current smoking	14487 (16.5%)	12990 (10.2%)
Body mass index		
<30	19707 (25.8%)	34354 (31.3%)
25-30	36,602 (48.0%)	51182 (46.6%)
>30	19,958 (26.2%)	24317 (22.1%)
Diabetes	12,451 (13.8%)	28784 (22.1%)

HRS = Health and Retirement Study, SHARE = Survey of Health, Aging and Retirement in Europe

Supplementary Table S4 Descriptive statistics of female participants in pooled HRS and SHARE by labor force status

	Working<br>(obs. = 93205)	Retired<br>(obs. = 146187)
Dental services use	51729 (55.5%)	104231 (71.3%)
Age, years		
Married	59.3 (6.5)	73.2 (8.6)
Education		
Low	17836 (19.1%)	55920 (38.3%)
Middle	34177 (36.7%)	52023 (35.6%)
High	41190 (44.2%)	38240 (26.2%)
Type of previous work		
No physically demanding work	69372 (74.4%)	-
Physically demanding work	23833 (25.6%)	-
Dental coverage	25331 (27.2%)	25736 (17.6%)
Current smoking	12481 (13.8%)	10784 (7.6%)
Body mass index		
<30	30089 (38.9%)	47674 (39.1%)
25-30	26391 (34.1%)	43680 (35.9%)
>30	20912 (27.0%)	30429 (25.0%)
Diabetes	9599 (10.3%)	27519 (18.8%)

HRS = Health and Retirement Study, SHARE = Survey of Health, Aging and Retirement in Europe

Supplementary Table S5 Descriptive statistics of observations by labor force status in Austria

	Working (obs. = 3040)	Retired (obs. = 9973)
Dental services use	3262 (57.2%)	7180 (72.0%)
Age, years	58.9 (6.0)	71.3 (8.3)
Men	1461 (48.1%)	4493 (45.1%)
Married	2034 (66.9%)	5791 (58.1%)
Education		
Low	397 (13.1%)	2,545 (25.5%)
Middle	1500 (49.3%)	5031 (50.4%)
High	1143 (37.6%)	2397 (24.0%)
Type of previous work		
No physically demanding work	2481 (81.6%)	-
Physically demanding work	559 (18.4%)	-
Dental coverage	675 (22.2%)	3265 (32.7%)
Current smoking	503 (16.5%)	827 (8.3%)
Body mass index		
<30	792 (40.9%)	2770 (38.1%)
25-30	781 (40.4%)	2919 (40.1%)
>30	362 (18.7%)	1587 (21.8%)
Diabetes	273 (9.0%)	1621 (16.3%)

Supplementary Table S6 Descriptive statistics of observations by labor force status in Belgium

	Working (obs. = 7154)	Retired (obs. = 12511)
Dental services use	3792 (53.0%)	8495 (67.9%)
Age, years	57.1 (5.3)	71.8 (8.2)
Men	3684 (51.5%)	6480 (51.8%)
Married	4849 (67.8%)	7880 (63.0%)
Education		
Low	1823 (25.5%)	5652 (45.2%)
Middle	2170 (30.3%)	3054 (24.4%)
High	3161 (44.2%)	3805 (30.4%)
Type of previous work		
No physically demanding work	5744 (80.3%)	-
Physically demanding work	1410 (19.7%)	-
Dental coverage	2334 (32.6%)	7089 (56.7%)
Current smoking	1093 (15.3%)	1038 (8.3%)
Body mass index		
<30	2176 (40.8%)	3746 (38.5%)
25-30	2140 (40.2%)	4062 (41.8%)
>30	1011 (19.0%)	1915 (19.7%)
Diabetes	534 (7.5%)	1859 (14.9%)

Supplementary Table S7 Descriptive statistics of observations by labor force status in Bulgaria

	Working (obs. = 737)	Retired (obs. = 1785)
Dental services use	186 (25.3%)	803 (45.0%)
Age, years	58.2 (5.8)	71.8 (7.5)
Men	354 (48.0%)	723 (40.5%)
Married	532 (72.2%)	1055 (59.1%)
Education		
Low	106 (14.4%)	651 (36.5%)
Middle	443 (60.1%)	881 (49.4%)
High	188 (25.5%)	253 (14.2%)
Type of previous work		
No physically demanding work	737 (100.0%)	-
Physically demanding work	0 (0%)	-
Dental coverage	0 (0%)	0 (0%)
Current smoking	72 (9.8%)	56 (3.1%)
Body mass index		
<30	231 (32.4%)	529 (31.0%)
25-30	313 (43.8%)	759 (44.5%)
>30	170 (23.8%)	417 (24.5%)
Diabetes	51 (6.9%)	310 (17.4%)

Supplementary Table S8 Descriptive statistics of observations by labor force status in Croatia

	Working (obs. = 1149)	Retired (obs. = 3656)
Dental services use	381 (33.2%)	1729 (47.3%)
Age, years	57.7 (4.5)	69.4 (7.9)
Men	581 (50.6%)	1,808 (49.5%)
Married	979 (85.2%)	2,673 (73.1%)
Education		
Low	389 (33.9%)	2,245 (61.4%)
Middle	446 (38.8%)	808 (22.1%)
High	314 (27.3%)	603 (16.5%)
Type of previous work		
No physically demanding work	1078 (93.8%)	-
Physically demanding work	71 (6.2%)	-
Dental coverage	0 (0%)	0 (0%)
Current smoking	232 (20.2%)	391 (10.7%)
Body mass index		
<30	359 (31.7%)	946 (26.5%)
25-30	546 (48.2%)	1657 (46.4%)
>30	228 (20.1%)	969 (27.1%)
Diabetes	84 (7.3%)	652 (17.8%)

Supplementary Table S9 Descriptive statistics of observations by labor force status in Cyprus

	Working (obs. = 317)	Retired (obs. =1153)
Dental services use	113 (35.7%)	601 (52.1%)
Age, years	57.8 (5.3)	75.1 (7.7)
Men	151 (47.6%)	502 (43.5%)
Married	279 (88.0%)	806 (69.9%)
Education		
Low	85 (26.8%)	716 (62.1%)
Middle	146 (46.1%)	291 (25.2%)
High	86 (27.1%)	146 (12.7%)
Type of previous work		
No physically demanding work	317 (100.0%)	-
Physically demanding work	0 (0 %)	-
Dental coverage	0 (0%)	0 (0%)
Current smoking	19 (6.0%)	44 (3.8%)
Body mass index		
<30	110 (35.5%)	318 (31.6%)
25-30	134 (43.2%)	463 (46.0%)
>30	66 (21.3%)	225 (22.4%)
Diabetes	26 (8.2%)	267 (23.2%)

Supplementary Table S10 Descriptive statistics of observations by labor force status in Czech Republic

	Working (obs. = 5468)	Retired (obs. = 13518)
Dental services use	3133 (57.3%)	10111 (74.8%)
Age, years	60.2 (6.5)	71.3 (7.7)
Men	2620 (47.9%)	5142 (38.0%)
Married	3807 (69.6%)	8149 (60.3%)
Education		
Low	1653 (30.2%)	6046 (44.7%)
Middle	2729 (49.9%)	5969 (44.2%)
High	1086 (19.9%)	1503 (11.1%)
Type of previous work		
No physically demanding work	4526 (82.8%)	-
Physically demanding work	942 (17.2%)	-
Dental coverage	0 (0%)	0 (0%)
Current smoking	927 (17.0%)	1386 (10.3%)
Body mass index		
<30	1002 (27.5%)	2409 (24.6%)
25-30	1624 (44.5%)	4314 (44.0%)
>30	1021 (28.0%)	3071 (31.4%)
Diabetes	654 (12.0%)	3648 (27.0%)

Supplementary Table S11 Descriptive statistics of observations by labor force status in Denmark

	Working (obs. = 7996)	Retired (obs. = 7884)
Dental services use	7028 (87.9%)	6228 (79.0%)
Age, years	58.9 (6.1)	73.3 (7.7)
Men	4096 (51.2%)	3413 (43.3%)
Married	5898 (73.8%)	5041 (63.9%)
Education		
Low	821 (10.3%)	2035 (25.8%)
Middle	3053 (38.2%)	3188 (40.4%)
High	4122 (51.6%)	2661 (33.8%)
Type of previous work		
No physically demanding work	6424 (80.3%)	-
Physically demanding work	1572 (19.7%)	-
Dental coverage	0 (0%)	0 (0%)
Current smoking	1133 (14.2%)	1050 (13.3%)
Body mass index		
<30	2592 (43.4%)	2804 (46.8%)
25-30	2378 (39.8%)	2312 (38.6%)
>30	999 (16.7%)	879 (14.7%)
Diabetes	453 (5.7%)	908 (11.5%)

Supplementary Table S12 Descriptive statistics of observations by labor force status in Estonia

	Working (obs. = 6936)	Retired (obs. = 11042)
Dental services use	2129 (30.7%)	5576 (50.5%)
Age, years	60.9 (6.7)	74.8 (7.6)
Men	2916 (42.0%)	4039 (36.6%)
Married	4437 (64.0%)	5855 (53.0%)
Education		
Low	728 (10.5%)	3,960 (35.9%)
Middle	3754 (54.1%)	5043 (45.7%)
High	2454 (35.4%)	2039 (18.5%)
Type of previous work		
No physically demanding work	5693 (82.1%)	-
Physically demanding work	1243 (17.9%)	-
Dental coverage	0 (0%)	0 (0%)
Current smoking	850 (12.3%)	618 (5.6%)
Body mass index		
<30	1420 (29.8%)	2374 (31.1%)
25-30	1935 (40.6%)	2927 (38.3%)
>30	1409 (29.6%)	2343 (30.7%)
Diabetes	703 (10.1%)	2347 (21.3%)

Supplementary Table S13 Descriptive statistics of observations by labor force status in Finland

	Working (obs. = 1053)	Retired (obs. = 1902)
Dental services use	680 (64.6%)	1360 (71.5%)
Age, years	58.3 (5.4)	72.1 (7.5)
Men	470 (44.6%)	915 (48.1%)
Married	758 (72.0%)	1287 (67.7%)
Education		
Low	115 (10.9%)	729 (38.3%)
Middle	371 (35.2%)	579 (30.4%)
High	567 (53.8%)	594 (31.2%)
Type of previous work		
No physically demanding work	1053 (100.0%)	1902 (100.0%)
Physically demanding work	0%	0%
Dental coverage	0%	0%
Current smoking	49 (4.7%)	56 (2.9%)
Body mass index		
<30	363 (35.0%)	632 (33.8%)
25-30	425 (41.0%)	816 (43.7%)
>30	249 (24.0%)	420 (22.5%)
Diabetes	104 (9.9%)	354 (18.6%)

Supplementary Table S14 Descriptive statistics of observations by labor force status in France

	Working (obs. = 5496)	Retired (obs. = 11959)
Dental services use	2666 (48.5%)	6757 (56.5%)
Age, years	57.4 (5.2)	72.5 (8.4)
Men	2526 (46.0%)	5456 (45.6%)
Married	3738 (68.0%)	7300 (61.0%)
Education		
Low	1339 (24.4%)	5986 (50.1%)
Middle	2366 (43.0%)	3749 (31.3%)
High	1791 (32.6%)	2224 (18.6%)
Type of previous work		
No physically demanding work	4190 (76.2%)	-
Physically demanding work	1306 (23.8%)	-
Dental coverage	2016 (36.7%)	5647 (47.2%)
Current smoking	837 (15.2%)	852 (7.1%)
Body mass index		
<30	1946 (47.3%)	3821 (41.6%)
25-30	1490 (36.2%)	3553 (38.7%)
>30	675 (16.4%)	1801 (19.6%)
Diabetes	385 (7.0%)	1846 (15.4%)

Supplementary Table S15 Descriptive statistics of observations by labor force status in Germany

	Working (obs. = 8473)	Retired (obs. = 10990)
Dental services use	6608 (78.0%)	9155 (83.3%)
Age, years	58.7 (6.0)	72.5 (7.3)
Men	4085 (48.2%)	5616 (51.1%)
Married	6456 (76.2%)	7835 (71.3%)
Education		
Low	550 (6.5%)	1596 (14.5%)
Middle	4891 (57.7%)	6204 (56.5%)
High	3032 (35.8%)	3190 (29.0%)
Type of previous work		
No physically demanding work	6710 (79.2%)	-
Physically demanding work	1763 (20.8%)	-
Dental coverage	2050 (24.2%)	3944 (35.9%)
Current smoking	1266 (14.9%)	880 (8.0%)
Body mass index		
<30	2321 (38.5%)	2967 (35.3%)
25-30	2501 (41.5%)	3624 (43.1%)
>30	1211 (20.1%)	1811 (21.6%)
Diabetes	762 (9.0%)	2143 (19.5%)

Supplementary Table S16 Descriptive statistics of observations by labor force status in Greece

	Working (obs. = 4246)	Retired (obs. = 7491)
Dental services use	1287 (30.3%)	3476 (46.4%)
Age, years	58.5 (5.8)	72.2 (8.7)
Men	2584 (60.9%)	4460 (59.5%)
Married	3317 (78.1%)	5308 (70.9%)
Education		
Low	1473 (34.7%)	4471 (59.7%)
Middle	1471 (34.6%)	1641 (21.9%)
High	1302 (30.7%)	1379 (18.4%)
Type of previous work		
No physically demanding work	2508 (59.1%)	-
Physically demanding work	1738 (40.9%)	-
Dental coverage	0 (0%)	0 (0%)
Current smoking	1313 (30.9%)	1115 (14.9%)
Body mass index		
<30	1193 (28.4%)	2062 (27.9%)
25-30	2235 (53.1%)	3814 (51.6%)
>30	779 (18.5%)	1518 (20.5%)
Diabetes	319 (7.5%)	1338 (17.9%)

Supplementary Table S17 Descriptive statistics of observations by labor force status in Hungary

	Working (obs. = 377)	Retired (obs. = 1793)
Dental services use	78 (21.2%)	570 (31.8%)
Age, years	60.8 (4.5)	71.4 (7.2)
Men	182 (48.3%)	678 (37.8%)
Married	262 (69.5%)	1,028 (57.3%)
Education		
Low	38 (10.1%)	539 (30.1%)
Middle	245 (65.0%)	983 (54.8%)
High	94 (24.9%)	271 (15.1%)
Type of previous work		
No physically demanding work	338 (89.7%)	-
Physically demanding work	39 (10.3%)	-
Dental coverage	0 (0%)	0 (0%)
Current smoking	43 (11.4%)	75 (4.2%)
Body mass index		
<30	96 (26.2%)	542 (30.8%)
25-30	146 (39.8%)	679 (38.6%)
>30	125 (34.1%)	536 (30.5%)
Diabetes	61 (16.2%)	511 (28.5%)

Supplementary Table S18 Descriptive statistics of observations by labor force status in Ireland

	Working (obs. = 395)	Retired (obs. = 336)
Dental services use	147 (37.2%)	169 (50.4%)
Age, years	58.0 (5.7)	71.7 (8.0)
Men	222 (56.2%)	192 (57.1%)
Married	297 (75.2%)	201 (59.8%)
Education		
Low	120 (30.4%)	136 (40.5%)
Middle	75 (19.0%)	57 (17.0%)
High	200 (50.6%)	143 (42.6%)
Type of previous work		
No physically demanding work	178 (45.1%)	-
Physically demanding work	217 (54.9%)	-
Dental coverage	0 (0%)	0 (0%)
Current smoking	68 (17.2%)	49 (14.6%)
Body mass index		
<30	140 (35.9%)	120 (36.1%)
25-30	138 (35.4%)	113 (34.0%)
>30	112 (28.7%)	99 (29.8%)
Diabetes	19 (4.8%)	44 (13.1%)

Supplementary Table S19 Descriptive statistics of observations by labor force status in Israel

	Working (obs. = 4461)	Retired (obs. = 4962)
Dental services use	1699 (38.1%)	2560 (51.6%)
Age, years	61.7 (6.7)	73.8 (8.2)
Men	2274 (51.0%)	2361 (47.6%)
Married	3552 (79.6%)	3413 (68.8%)
Education		
Low	1069 (24.0%)	1659 (33.4%)
Middle	1542 (34.6%)	1760 (35.5%)
High	1850 (41.5%)	1543 (31.1%)
Type of previous work		
No physically demanding work	3503 (78.5%)	-
Physically demanding work	958 (21.5%)	-
Dental coverage	0 (0%)	0 (0%)
Current smoking	622 (13.9%)	303 (6.1%)
Body mass index		
<30	1222 (38.9%)	1314 (35.0%)
25-30	1385 (44.0%)	1626 (43.3%)
>30	538 (17.1%)	812 (21.6%)
Diabetes	823 (18.4%)	1563 (31.5%)

Supplementary Table S20 Descriptive statistics of observations by labor force status in Italy

	Working (obs. = 4997)	Retired (obs. = 11210)
Dental services use	1604 (32.1%)	4753 (42.4%)
Age, years	57.8 (5.4)	71.9 (7.8)
Men	2803 (56.1%)	6511 (58.1%)
Married	3986 (79.8%)	8529 (76.1%)
Education		
Low	2344 (46.9%)	8414 (75.1%)
Middle	1789 (35.8%)	2101 (18.7%)
High	864 (17.3%)	695 (6.2%)
Type of previous work		
No physically demanding work	3601 (72.1%)	-
Physically demanding work	1396 (27.9%)	-
Dental coverage	0 (0%)	0 (0%)
Current smoking	801 (16.0%)	979 (8.7%)
Body mass index		
<30	1615 (42.4%)	3339 (38.1%)
25-30	1693 (44.4%)	4015 (45.8%)
>30	505 (13.2%)	1407 (16.1%)
Diabetes	339 (6.8%)	2071 (18.5%)

Supplementary Table S21 Descriptive statistics of observations by labor force status in Latvia

	Working (obs. = 724)	Retired (obs. = 1464)
Dental services use	199 (27.5%)	722 (49.3%)
Age, years	58.0 (5.1)	73.5 (7.6)
Men	326 (45.0%)	475 (32.4%)
Married	495 (68.4%)	674 (46.0%)
Education		
Low	28 (3.9%)	377 (25.8%)
Middle	459 (63.4%)	757 (51.7%)
High	237 (32.7%)	330 (22.5%)
Type of previous work		
No physically demanding work	724 (100.0%)	-
Physically demanding work	0 (0%)	-
Dental coverage	0 (0%)	0 (0%)
Current smoking	36 (5.0%)	57 (3.9%)
Body mass index		
<30	177 (24.8%)	370 (26.1%)
25-30	288 (40.3%)	586 (41.4%)
>30	249 (34.9%)	459 (32.4%)
Diabetes	47 (6.5%)	204 (13.9%)

Supplementary Table S22 Descriptive statistics of observations by labor force status in Lithuania

	Working (obs. = 1075)	Retired (obs. = 1793)
Dental services use	396 (36.8%)	961 (53.6%)
Age, years	58.2 (5.5)	74.5 (7.7)
Men	425 (39.5%)	605 (33.7%)
Married	723 (67.3%)	858 (47.9%)
Education		
Low	35 (3.3%)	540 (30.1%)
Middle	549 (51.1%)	684 (38.1%)
High	491 (45.7%)	569 (31.7%)
Type of previous work		
No physically demanding work	1075 (100.0%)	-
Physically demanding work	0 (0%)	-
Dental coverage	0 (0%)	0 (0%)
Current smoking	92 (8.6%)	79 (4.4%)
Body mass index		
<30	302 (28.1%)	492 (28.0%)
25-30	466 (43.4%)	739 (42.0%)
>30	305 (28.4%)	528 (30.0%)
Diabetes	66 (6.1%)	214 (11.9%)

Supplementary Table S23 Descriptive statistics of observations by labor force status in Luxembourg

	Working (obs. = 1292)	Retired (obs. = 2632)
Dental services use	937 (72.5%)	2258 (85.8%)
Age, years	57.2 (4.7)	69.6 (7.7)
Men	628 (48.6%)	1635 (62.1%)
Married	1026 (79.4%)	1977 (75.1%)
Education		
Low	384 (29.7%)	1138 (43.2%)
Middle	496 (38.4%)	1082 (41.1%)
High	412 (31.9%)	412 (15.7%)
Type of previous work		
No physically demanding work	1199 (92.8%)	-
Physically demanding work	93 (7.2%)	-
Dental coverage	0 (0%)	0 (0%)
Current smoking	137 (10.6%)	196 (7.4%)
Body mass index		
<30	380 (44.5%)	641 (34.4%)
25-30	322 (37.7%)	726 (38.9%)
>30	151 (17.7%)	498 (26.7%)
Diabetes	72 (5.6%)	475 (18.0%)

Supplementary Table S24 Descriptive statistics of observations by labor force status in Malta

	Working (obs. = 367)	Retired (obs. = 861)
Dental services use	134 (36.4%)	437 (50.7%)
Age, years	58.6 (4.8)	71.1 (7.2)
Men	233 (63.5%)	640 (74.3%)
Married	312 (85.0%)	690 (80.1%)
Education		
Low	60 (16.3%)	453 (52.6%)
Middle	262 (71.4%)	337 (39.1%)
High	45 (12.3%)	71 (8.2%)
Type of previous work		
No physically demanding work	367 (100.0%)	-
Physically demanding work	0 (0%)	-
Dental coverage	0 (0%)	
Current smoking	25 (6.8%)	43 (5.0%)
Body mass index		
<30	62 (18.1%)	152 (20.0%)
25-30	128 (37.3%)	283 (37.2%)
>30	153 (44.6%)	326 (42.8%)
Diabetes	51 (13.9%)	200 (23.2%)

Supplementary Table S25 Descriptive statistics of observations by labor force status in the Netherland

	Working (obs. = 4043)	Retired (obs. 4599)
Dental services use	2490 (61.6%)	3665 (79.7%)
Age, years	58.4 (6.1)	72.7 (7.1)
Men	2131 (52.7%)	2621 (57.0%)
Married	3122 (77.2%)	3284 (71.4%)
Education		
Low	1394 (34.5%)	2431 (52.9%)
Middle	1173 (29.0%)	1086 (23.6%)
High	1476 (36.5%)	1082 (23.5%)
Type of previous work		
No physically demanding work	3188 (78.9%)	-
Physically demanding work	855 (21.1%)	-
Dental coverage	1587 (39.3%)	2123 (46.2%)
Current smoking	841 (20.8%)	640 (13.9%)
Body mass index		
<30	1128 (45.1%)	1156 (41.6%)
25-30	1051 (42.1%)	1234 (44.4%)
>30	320 (12.8%)	387 (13.9%)
Diabetes	246 (6.1%)	605 (13.2%)

Supplementary Table S26 Descriptive statistics of observations by labor force status in Poland

	Working (obs. = 2686)	Retired (obs. = 6349)
Dental services use	610 (22.7%)	2889 (45.5%)
Age, years	57.3 (5.0)	71.0 (8.5)
Men	1390 (51.7%)	2621 (41.3%)
Married	2146 (79.9%)	4424 (69.7%)
Education		
Low	327 (12.2%)	2797 (44.1%)
Middle	1869 (69.6%)	3005 (47.3%)
High	490 (18.2%)	547 (8.6%)
Type of previous work		
No physically demanding work	2253 (83.9%)	-
Physically demanding work	433 (16.1%)	-
Dental coverage	0 (0%)	0 (0%)
Current smoking	433 (16.1%)	565 (8.9%)
Body mass index		
<30	873 (32.7%)	1823 (29.5%)
25-30	1,133 (42.4%)	2556 (41.4%)
>30	664 (24.9%)	1798 (29.1%)
Diabetes	246 (9.2%)	1330 (20.9%)

Supplementary Table S27 Descriptive statistics of observations by labor force status in Portugal

	Working (obs. = 516)	Retired (obs. = 2247)
Dental services use	71 (13.8%)	440 (19.6%)
Age, years	57.1 (4.8)	69.7 (8.2)
Men	279 (54.1%)	991 (44.1%)
Married	436 (84.5%)	1446 (64.4%)
Education		
Low	134 (26.0%)	1149 (51.1%)
Middle	319 (61.8%)	1003 (44.6%)
High	63 (12.2%)	95 (4.2%)
Type of previous work		
No physically demanding work	516 (100.0%)	-
Physically demanding work	0 (0%)	-
Dental coverage	0 (0%)	0 (0%)
Current smoking	59 (11.4%)	107 (4.8%)
Body mass index		
<30	149 (29.0%)	627 (28.8%)
25-30	220 (42.9%)	884 (40.7%)
>30	144 (28.1%)	663 (30.5%)
Diabetes	26 (5.0%)	327 (14.6%)

Supplementary Table S28 Descriptive statistics of observations by labor force status in Rumania

	Working (obs. = 1281)	Retired (obs. = 1534)
Dental services use	676 (52.8%)	1088 (70.9%)
Age, years	56.4 (3.9)	68.1 (6.8)
Men	619 (48.3%)	708 (46.2%)
Married	1066 (83.2%)	1078 (70.3%)
Education		
Low	24 (1.9%)	266 (17.3%)
Middle	1060 (82.7%)	1174 (76.5%)
High	197 (15.4%)	94 (6.1%)
Type of previous work		
No physically demanding work	1281 (100.0%)	-
Physically demanding work	0 (0%)	-
Dental coverage	0 (0%)	0 (0%)
Current smoking	83 (6.5%)	70 (4.6%)
Body mass index		
<30	407 (32.0%)	414 (27.6%)
25-30	606 (47.7%)	694 (46.2%)
>30	258 (20.3%)	393 (26.2%)
Diabetes	31 (2.4%)	221 (14.4%)

Supplementary Table S29 Descriptive statistics of observations by labor force status in Slovakia

	Working (obs. = 1281)	Retired (obs. = 1534)
Dental services use	676 (52.8%)	1088 (70.9%)
Age, years	56.4 (3.9)	68.1 (6.8)
Men	619 (48.3%)	708 (46.2%)
Married	1066 (83.2%)	1078 (70.3%)
Education		
Low	24 (1.9%)	266 (17.3%)
Middle	1060 (82.7%)	1174 (76.5%)
High	197 (15.4%)	94 (6.1%)
Type of previous work		
No physically demanding work	1281 (100.0%)	
Physically demanding work	0 (0%)	
Dental coverage	0 (0%)	
Current smoking	83 (6.5%)	70 (4.6%)
Body mass index		
<30	407 (32.0%)	414 (27.6%)
25-30	606 (47.7%)	694 (46.2%)
>30	258 (20.3%)	393 (26.2%)
Diabetes	31 (2.4%)	221 (14.4%)

Supplementary Table S30 Descriptive statistics of observations by labor force status in Slovenia

	Working (obs. = 2114)	Retired (obs. = 9606)
Dental services use	890 (42.1%)	5715 (59.5%)
Age, years	57.3 (4.6)	70.7 (8.3)
Men	981 (46.4%)	4263 (44.4%)
Married	1640 (77.6%)	6687 (69.6%)
Education		
Low	269 (12.7%)	3177 (33.1%)
Middle	1141 (54.0%)	5020 (52.3%)
High	704 (33.3%)	1409 (14.7%)
Type of previous work		
No physically demanding work	1846 (87.3%)	-
Physically demanding work	268 (12.7%)	-
Dental coverage	0 (0%)	0 (0%)
Current smoking	252 (11.9%)	542 (5.6%)
Body mass index		
<30	494 (32.1%)	2169 (28.9%)
25-30	708 (45.9%)	3435 (45.8%)
>30	339 (22.0%)	1889 (25.2%)
Diabetes	213 (10.1%)	1780 (18.5%)

Supplementary Table S31 Descriptive statistics of observations by labor force status in Spain

	Working (obs. = 5097)	Retired (obs. = 10727)
Dental services use	1300 (25.5%)	4044 (37.7%)
Age, years	58.5 (5.2)	74.4 (8.3)
Men	2710 (53.2%)	6845 (63.8%)
Married	3985 (78.2%)	7874 (73.4%)
Education		
Low	3027 (59.4%)	9011 (84.0%)
Middle	1016 (19.9%)	780 (7.3%)
High	1054 (20.7%)	936 (8.7%)
Type of previous work		
No physically demanding work	4034 (79.1%)	-
Physically demanding work	1063 (20.9%)	-
Dental coverage	0 (0%)	0 (0%)
Current smoking	732 (14.4%)	652 (6.1%)
Body mass index		
<30	1196 (35.7%)	2261 (31.2%)
25-30	1533 (45.8%)	3545 (48.8%)
>30	617 (18.4%)	1451 (20.0%)
Diabetes	472 (9.3%)	2758 (25.7%)

Supplementary Table S32 Descriptive statistics of observations by labor force status in Sweden

	Working (obs. = 7958)	Retired (obs. =10890)
Dental services use	6430 (80.8%)	9158 (84.1%)
Age, years	61.8 (6.7)	74.4 (7.6)
Men	3883 (48.8%)	4926 (45.2%)
Married	5566 (69.9%)	7064 (64.9%)
Education		
Low	2042 (25.7%)	5477 (50.3%)
Middle	2830 (35.6%)	2943 (27.0%)
High	3086 (38.8%)	2470 (22.7%)
Type of previous work		
No physically demanding work	6399 (80.4%)	-
Physically demanding work	1559 (19.6%)	-
Dental coverage	0 (0%)	0 (0%)
Current smoking	839 (10.5%)	751 (6.9%)
Body mass index		
<30	2566 (43.3%)	3811 (45.7%)
25-30	2445 (41.3%)	3250 (39.0%)
>30	916 (15.5%)	1275 (15.3%)
Diabetes	681 (8.6%)	1529 (14.0%)

Supplementary Table S33 Descriptive statistics of observations by labor force status in Switzerland

	Working (obs. = 5337)	Retired (obs. = 5751)
Dental services use	3827 (71.7%)	4647 (80.8%)
Age, years	61.0 (6.5)	74.4 (7.3)
Men	2677 (50.2%)	2717 (47.2%)
Married	3697 (69.3%)	3580 (62.3%)
Education		
Low	829 (15.5%)	1658 (28.8%)
Middle	3378 (63.3%)	3392 (59.0%)
High	1130 (21.2%)	701 (12.2%)
Type of previous work		
No physically demanding work	4343 (81.4%)	-
Physically demanding work	994 (18.6%)	-
Dental coverage	0 (0%)	0 (0%)
Current smoking	778 (14.6%)	502 (8.7%)
Body mass index		
<30	1942 (50.2%)	2044 (46.2%)
25-30	1395 (36.0%)	1767 (39.9%)
>30	533 (13.8%)	618 (14.0%)
Diabetes	342 (6.4%)	652 (11.3%)

Supplementary Table S34 Descriptive statistics of observations by labor force status in United States

	Working (obs. = 88110)	Retired (obs. = 104029)
Dental services use	53042 (60.2%)	72508 (69.7%)
Age, years	60.9 (7.3)	74.4 (8.8)
Men	42738 (48.5%)	47460 (45.6%)
Married	59660 (67.7%)	57720 (55.5%)
Education		
Low	15575 (17.7%)	29004 (27.9%)
Middle	24099 (27.4%)	32771 (31.5%)
High	48421 (55.0%)	42233 (40.6%)
Type of previous work		
No physically demanding work	58594 (66.5%)	-
Physically demanding work	29516 (33.5%)	-
Dental coverage	39042 (44.3%)	26217 (25.2%)
Current smoking	12823 (15.5%)	9843 (10.1%)
Body mass index		
<30	22342 (27.4%)	34818 (36.3%)
25-30	32543 (39.9%)	36801 (38.4%)
>30	26643 (32.7%)	24292 (25.3%)
Diabetes	13882 (15.8%)	24059 (23.1%)

Supplementary Table S35 Official and early retirement age by country

		Men		Women	
Country	Year	ORA	ERA	ORA	ERA
Austria	2018	-	65	-	60
Belgium	2018	63	65	63	65
Bulgaria	2018	63.08	64.08	60.17	61.17
Croatia	2018	60	65	57	62
Cyprus	2018	63	65	63	65
Czech Republic	2018	60	63.16	59.66	62.66
Denmark	2018	-	65	-	65
Great Britain	2018	-	65	-	65
Estonia	2018	60.5	63.5	60.5	63.5
Finland	2018	63	65	63	65
France	2018	62	67	62	67
Germany	2018	63	65.58	63	65.58
Greece	2018	62	67	62	67
Hungary	2018	-	63.5	-	63.5
Ireland	2018	-	66	-	66
Israel	2018	-	67	-	62
Italy	2018	62	66.58	62	66.58
Latvia	2018	61.25	63.25	61.25	63.25
Lithuania	2018	58.67	63.67	57.33	62.33
Luxembourg	2018	57	65	57	65
Malta	2018	61	62	61	62
Netherlands	2018	-	66	-	66
Poland	2018	-	65	-	60
Portugal	2018	60	66.33	60	66,33
Romania	2018	60	65	55.92	60.92
Slovakia	2018	60.42	62.42	60.42	62.42
Slovenia	2018	60	65	59.67	64
Spain	2018	61.5	65.5	61.5	65.5
Sweden	2018	61	65	61	65
Switzerland	2018	63	65	62	64

United States	2018	62	66	62	66

Source: OECD. Pensions at a Glance 2021. Paris: OECD Publishing, 2021. The United States Social Security Administration. Social Security Programs Throughout the World. https://www.ssa.gov/policy/docs/progdesc/ssptw/ (accessed Aug 11, 2023).
ORA = official retirement age, ERA = early retirement age

Supplementary Table S36 Fixed-effects ordered logit model and fixed-effects control function ordered logit model estimates for retirement and self-rated oral health based on ELSA panel data

	e^{β}	95% CI	*P*-value
Fixed-effects ordered logit model	0.680	0.610–0.759	5.2e-12
Fixed-effects control function ordered logit model	0.598	0.370–0.966	3.6e-02

ELSA = English Longitudinal Study on Aging

Supplementary Table S37 Fixed-effects Poisson model and fixed-effects control function Poisson model estimates for retirement and dental services use based on HRS+SHARE panel data

	e^{β}	95% CI	*P*-value
Fixed-effects Poisson model	1.069	1.041–1.097	6.6e-07
Fixed-effects control function Poisson model	1.325	1.242–1.413	1.4e-17

HRS = Health and Retirement Study, SHARE = Survey of Health, Aging and Retirement in Europe
